# Role of Murine Intestinal Interleukin-1 Receptor 1-Expressing Lymphoid Tissue Inducer-Like Cells in *Salmonella* Infection

**DOI:** 10.1371/journal.pone.0065405

**Published:** 2013-06-04

**Authors:** Vincent L. Chen, Neeraj K. Surana, Jinyou Duan, Dennis L. Kasper

**Affiliations:** 1 Department of Microbiology and Immunobiology, Harvard Medical School, Boston, Massachusetts, United States of America; 2 Division of Infectious Diseases, Boston Children’s Hospital, Boston, Massachusetts, United States of America; 3 College of Science, Northwest A&F University, Yangling, China; Massachusetts General Hospital, United States of America

## Abstract

Interleukin (IL)-1 signaling plays a critical role in intestinal immunology. Here, we report that the major population of intestinal lamina propria lymphocytes expressing IL-1 receptor 1 (IL-1R1) is the lymphoid tissue inducer (LTi)-like cell, a type of innate lymphoid cell. These cells are significant producers of IL-22, and this IL-22 production depends on IL-1R1 signaling. LTi-like cells are required for defense against *Salmonella enterica* serovar Typhimurium. Moreover, colonic LTi-like cell numbers depend on the presence of the intestinal microbiota. LTi-like cells require IL-1R1 for production of protective cytokines and confer protection in infectious colitis, and their cell numbers in the colon depend upon having a microbiome.

## Introduction

The mammalian immune system must respond robustly to intestinal pathogens without initiating inappropriate immune reactions against the estimated 10^14^ commensal intestinal bacteria [1], [2]. Abnormal host-bacterial interactions can result in inflammatory diseases such as inflammatory bowel disease or vulnerability to infection [3], [4]. While the host and bacterial factors involved in these interactions are beginning to be elucidated [5], [6], [7], [8], [9], [10], a more complete understanding of the different molecular and cellular factors involved in maintenance of intestinal homeostasis is necessary for the development of additional therapeutic agents targeted against disorders of the intestinal immune system.

Host-bacterial interactions are not limited to balancing protection against pathogens with tolerance to commensal bacteria: commensal bacteria also drive the development of the mammalian immune system. For example, *Bacteroides fragilis*, a member of the normal human microbiome, produces polysaccharide A, which regulates the systemic murine helper T cell (Th)1-Th2 balance [8] and induces the development of interleukin (IL)-10-producing regulatory T cells that protect against murine colitis and experimental autoimmune encephalomyelitis [9], [11]. Murine segmented filamentous bacterium (SFB) contributes to Th17 cell maturation [6] by stimulating dendritic cells to drive T cell differentiation through yet-uncharacterized mechanisms that may involve adhesion proteins, glycan utilization enzymes, or uptake of metals such as iron [12], [13]. Finally, *Clostridium* species induce the development of IL-10-producing regulatory T cells in the murine colon, which protect against chemically-induced colitis and suppress systemic immunoglobulin E responses [5].

Innate lymphoid cells (ILCs) are increasingly recognized as being vital to intestinal homeostasis [14], [15]. ILCs are found in large numbers in the intestine and also play a role in airway mucosal immunity [16]. ILCs are divided into three groups based on their cytokine production profiles [14]. Group 1 ILCs produce Th1 cytokines such as interferon (IFN)-γ, but not Th17 cytokines. The natural killer cell is the prototypic group 1 ILC. Group 2 ILCs produce Th2 cytokines such as IL-5 and IL-13. The ILCs most relevant to this paper are group 3 ILCs, which produce IL-22 and/or IL-17 and IFN-γ and express retinoic acid-related orphan receptor (ROR)γt. The best-characterized classes of group 3 ILCs are ILCs, which are NKp46^+^ cells that produce IL-22 and unlike conventional NK cells do not appear to have direct cytotoxic effects or produce IFN-γ [17], and lymphoid tissue inducer (LTi) cells [15].

LTi cells–named for their role in the generation of secondary lymphoid organs–are a critical component of the intestinal immune system. These cells, which are found in secondary lymphoid tissues and the intestines, are c-kit^+^ cluster of differentiation 127 (CD127)^+^ RORγt^+^ but negative for the lineage (Lin) markers CD3, CD11b, CD11c, or B220 [18], [19]. In secondary lymphoid tissues, LTi cells induce stromal cell expression of chemokine ligands that recruit T cells, B cells, and antigen presenting cells into spatially distinct regions by signaling through lymphotoxin-α_1_β_2_ [20] and help maintain memory CD4^+^ T cells [21]. In the intestines, they drive the formation of Peyer’s patches, as well as isolated lymphoid follicles in the gut that are important for secretory immunoglobulin A production, which in turn helps maintain a localized mucosal response to commensal bacteria without inducing systemic inflammation [22], [23], [24]. In addition, intestinal LTi cells protect against *Citrobacter rodentium* infection [25] and dextran sodium sulfate (DSS)-induced colitis [26] by secreting IL-22 [26], [27]. IL-22, in turn, has emerged as a major protective factor against various infectious and inflammatory diseases [28], [29], [30] by enhancing tissue healing, inducing anti-microbial peptide secretion, and recruiting phagocytes [31], [32]. As such, LTi cells play important roles both in preventing inappropriate immune responses against commensal bacteria and in defense against intestinal inflammatory conditions.

The role of the intestinal microbiota on group 3 ILCs remains controversial. Germ-free (GF) mice are varyingly found to have increased [19], [26] and decreased [17], [33] IL-22 production. The microbiota stimulates intestinal antigen presenting cells to produce IL-1 [34] and IL-23 [35], [36], which enhance ILC activity, but also induces epithelial cells to produce IL-25, which inhibits IL-22 production [26]. Likewise, GF mice may have either normal [37] or decreased [22] numbers of ILFs. While some studies have found that GF mice have fewer small-intestinal group 3 ILCs [17], [33], others have found that numbers are normal in GF mice [19], [26]. Importantly, the number of small-intestinal LTi cells is not affected by the microbiota: both specific pathogen-free (SPF) and GF mice have similar numbers [19]. We are not aware of previous studies investigating group 3 ILC numbers in the colon.

One major factor that regulates intestinal immunity is interleukin IL-1 [38]. Classically, IL-1 is known for enhancing a generalized immune response that includes proliferation and survival of naïve T cells, cytokine production and phagocytosis by macrophages, antigen presentation by dendritic cells, and recruitment of and oxidative burst by neutrophils [39], [40]. More recently, IL-1 has attracted renewed interest because of its effects on IL-17 and IL-22 secretion by lymphocytes. Dong and colleagues first demonstrated that IL-1, in synergy with IL-6 and IL-23, is necessary for both dendritic cell (DC)-mediated maturation of naïve T cells to Th17 cells and effector function of mature Th17 cells [41]. IL-1 also induces production of Th17 cytokines by other cell populations, including IL-17 by NKT cells [42] and IL-22 by ILCs [43], [44]. In the intestine, IL-1 plays an important role in driving both acute and chronic inflammation [38], [45], [46], [47].

Our laboratory recently demonstrated that IL-1 receptor 1 (IL-1R1) is important for driving the proinflammatory effects of IL-17-producing γδ T cells in both systemic and mucosal immunity [48]. To further explore the role of IL-1R1, we investigated IL-1R1 expression and function in the intestine. We identified high levels of IL-1R1 expression on intestinal lamina propria (LP) CD4^+^ LTi-like cells. IL-1R1 signaling to these cells results in enhanced production of IL-17 and IL-22 *in vitro*. These LTi-like cells are important mediators in the innate immune response against an intestinal pathogen, *Salmonella enterica* serovar Typhimurium (*S.* Typhimurium). Finally, we demonstrate that the number of IL-1R1^+^ LTi-like cells in the colon depends on the intestinal microbiome. Thus, this work has identified a signaling pathway in LTi-like cells with potential implications in the pathogenesis of defense against enteric pathogens and inflammatory bowel disease.

## Results

### Gut-associated IL-1R1^+^ LTi-like Cells are Found Primarily in the Intestinal Lamina Propria

**Figure 1:**
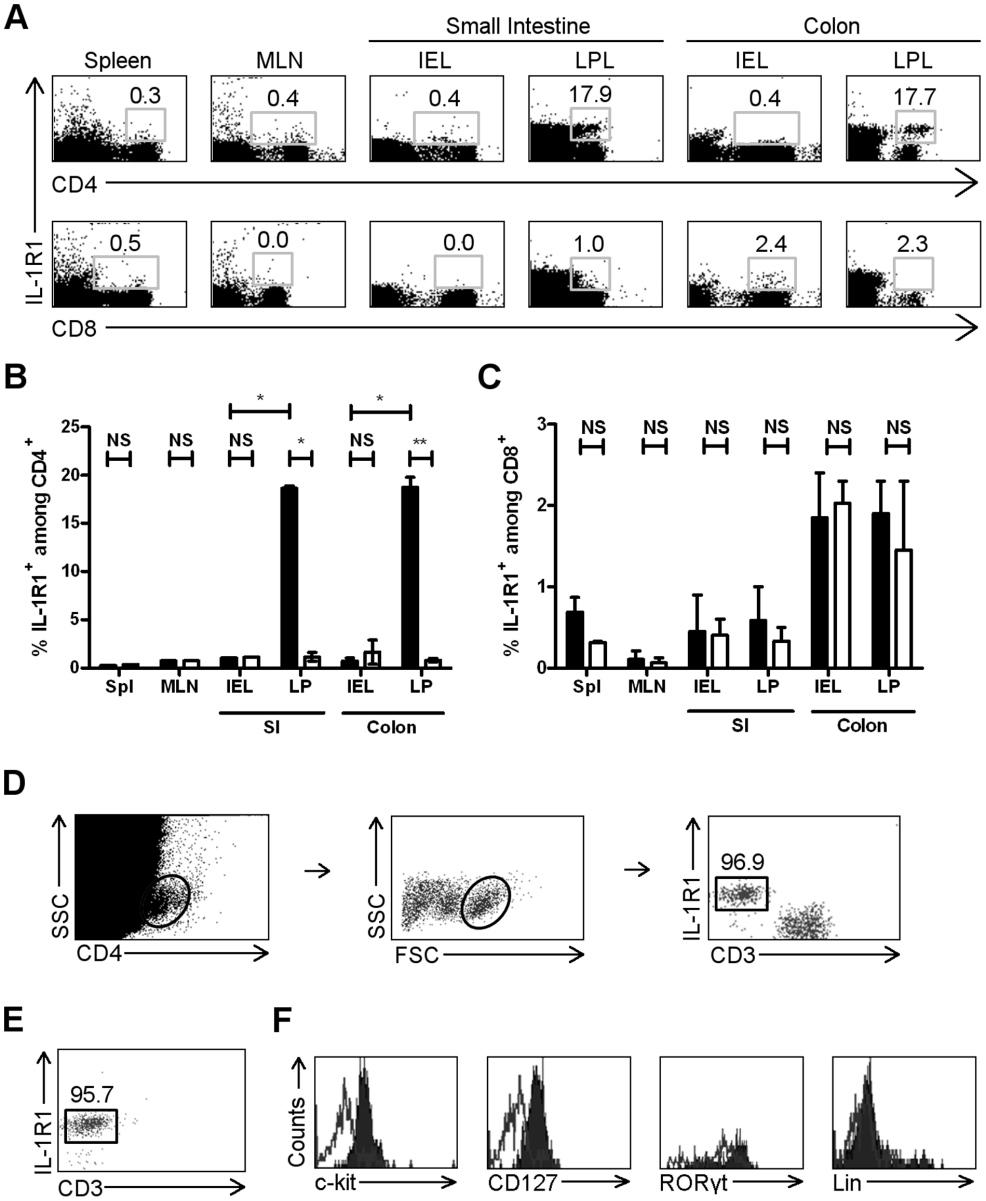
Gut-associated IL-1R1^+^ CD4^+^ Lin^−^ LTi-like cells are located predominantly in the intestinal lamina propria. (A) Representative FACS scatter plots depicting IL-1R1^+^ cells as a percentage of total CD4^+^ or CD8^+^ cells in spleen and gut-associated compartments of WT C57BL/6J mice. Numbers represent percentage of CD4^+^ or CD8^+^ cells expressing IL-1R1. (B and C) Bar graph depicting IL-1R1^+^ cells as a percentage of total CD4^+^ (B) or CD8^+^ (C) cells in spleen and gut-associated compartments of WT C57BL/6J mice. Bar represents median; error bar represents standard error. Filled bars represent anti-IL-1R1 flurophore-conjugated antibodies; empty bars represent isotype control antibodies. Spl, spleen; MLN, mesenteric lymph nodes; SI, small intestine; IEL, intraepithelial lymphocytes; LPL, lamina propria lymphocytes. Data collected from three independent experiments. (D) Gating scheme for IL-1R1^+^ CD4^+^ cells. SSC, side scatter. FSC, forward scatter. Number is the percent of CD4^+^ CD3^−^ lymphocytes that are IL-1R1^+^ in WT C57BL/6J mice. (E) Scatter plot depicting IL-1R1^+^ CD4^+^ cells in Rag1^−/−^ C57BL/6J mice. Number is the percent of CD4^+^ lymphocytes that are IL-1R1^+^ in Rag1^−/−^ mice. Gated on CD4^+^ lymphocytes as in (D). (F) FACS histograms demonstrating expression of c-kit, CD127, RORγt, and Lin in WT C57BL/6J mice. Plots are gated on IL-1R1^+^ CD4^+^ cells. Empty, isotype control; filled, antibody. *, p<0.05; **, p<0.01; NS, not significant.

Previous work in our laboratory demonstrated the importance of IL-1R1 signaling in the production of IL-17 by γδ T cells in mucosal and systemic immune compartments [48]. We were interested in extending this work by investigating IL-1R1 expression on T cells in the intestinal immune system. We isolated lymphocytes from the spleen and several gut-associated compartments of wild-type (WT) mice and analyzed them with fluorophore-conjugated antibodies against IL-1R1 in conjunction with antibodies against either CD4 or CD8. Flow cytometric analysis demonstrated that there are populations of CD4^+^ cells in the small-intestinal and colonic lamina propria (cLP) that express IL-1R1 (Fig. 1A and 1B). In contrast, virtually no CD8^+^ cells express IL-1R1 in the investigated compartments (Fig. 1A and 1C).

We reasoned that as the IL-1R1^+^ population constitutes a significant proportion of all CD4^+^ cells in the small-intestinal and colonic LPs, and a larger proportion in the LPs than the respective IEL compartments (Fig. 1B), they could have an important immunologic role, and began to further characterize them. Surprisingly, additional flow cytometric analysis utilizing the gating strategy depicted in Fig. 1D demonstrated that these IL-1R1^+^ CD4^+^ cells were CD3^−^; ∼97% of the cLP CD3^−^ CD4^+^ cells were IL-1R1^+^ whereas virtually none of the CD3^+^ CD4^+^ T cells expressed detectable levels of IL-1R1 (Fig. 1D). This IL-1R1^+^ CD4^+^ CD3^−^ population was present in the cLP of Rag1^−/−^ mice as well and accounted for over 95% of the CD4^+^ population in the colonic (Fig. 1E) and small-intestinal (data not shown) LP. Both colonic and small-intestinal IL-1R1^+^ CD4^+^ cells are c-kit^+^ CD127^+^ RORγt^+^ but do not express the Lin markers CD3, CD11b, CD11c, and B220 (Fig. 1F and data not shown); thus, they have the cell surface phenotype of LTi cells [51]. However, because we do not explicitly demonstrate lymphoid tissue inducer function, we refer to this population as “LTi-like” cells. Moreover, since essentially all Lin^−^ CD4^+^ cells in the intestinal LP are IL-1R1^+^, we continued to use the markers Lin^−^ CD4^+^ to identify intestinal IL-1R1^+^ LTi-like cells.

### Colonic LTi-like Cells are Significant Innate Producers of IL-22

**Figure 2:**
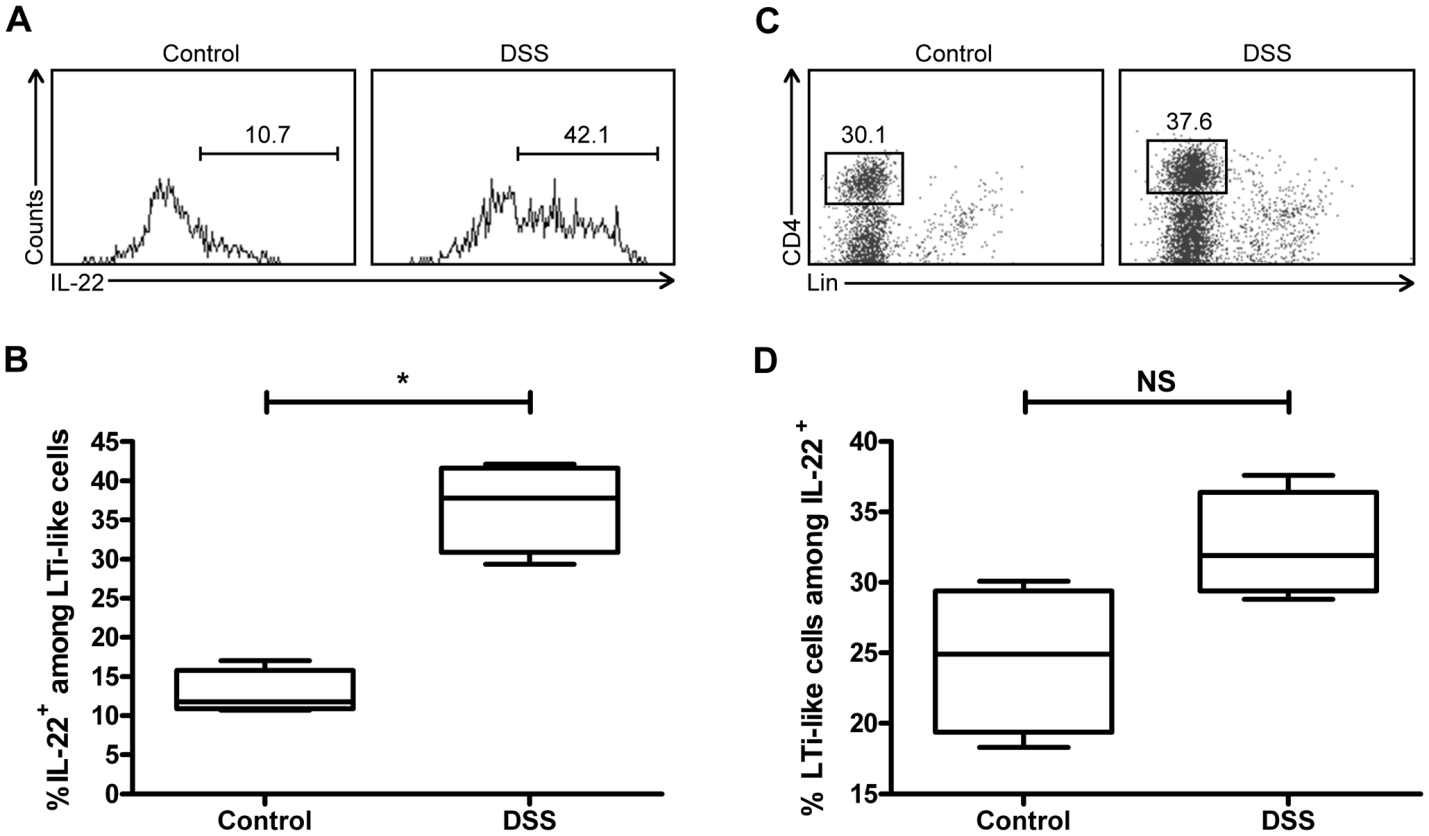
Colonic LTi-like cells are significant innate producers of IL-22. (A) Representative FACS histogram depicting percentage of CD4^+^ LTi-like cells that produce IL-22 under control conditions (*left panel*) and in DSS colitis (*right panel*) in Rag1^−/−^ C57BL/6J mice. (B) Graph summarizing percentage of CD4^+^ LTi-like cells producing IL-22. (C) Representative scatter plot depicting the phenotype of IL-22-producing lymphocytes under control conditions (*left panel*) and in DSS colitis (*right panel*) in Rag1^−/−^ C57BL/6J mice. Gated on IL-22^+^ lymphocytes. (D) Graph depicting percentage of IL-22-producing lymphocytes that are CD4^+^ LTi-like cells. All graphs represent three independent experiments. *, *p*<0.05; NS, not significant. For box and whisker plots, line represents median, box represents 25^th^ to 75^th^ percentile range, and whiskers represent range.

Given previous work demonstrating that LTi cells are a major source of IL-22 in the small intestine [26], we sought to determine whether colonic LTi-like cells are also a major producer of IL-22 under both healthy and inflammatory conditions. We induced colitis using the well-characterized DSS model, which is a chemical colitis mediated primarily by innate immune cells [30]. Rag1^−/−^ mice were used because the adaptive immune system suppresses the function of LTi-like cells [26] and we were interested primarily in the contribution of LTi-like cells to innate immunity. DSS colitis in Rag1^−/−^ mice induces colonic LTi-like cells to produce IL-22: the percentage of LTi-like cells producing IL-22 increases from ∼13% in healthy (control) conditions to ∼37% in inflammatory (DSS) conditions (Fig. 2A and 2B). In addition, colonic LTi-like cells constitute a substantial proportion of the IL-22-producing cells in the cLP of Rag1^−/−^ mice (Fig. 2C and 2D), both in health and in the setting of colitis. Overall, these data demonstrate that cLP CD4^+^ LTi-like cells are important innate producers of IL-22 and upregulate IL-22 production in the setting of intestinal inflammation.

### Intestinal LTi-like Cells Require IL-1R1 Signaling for IL-23-induced Production of IL-17 and IL-22

**Figure 3:**
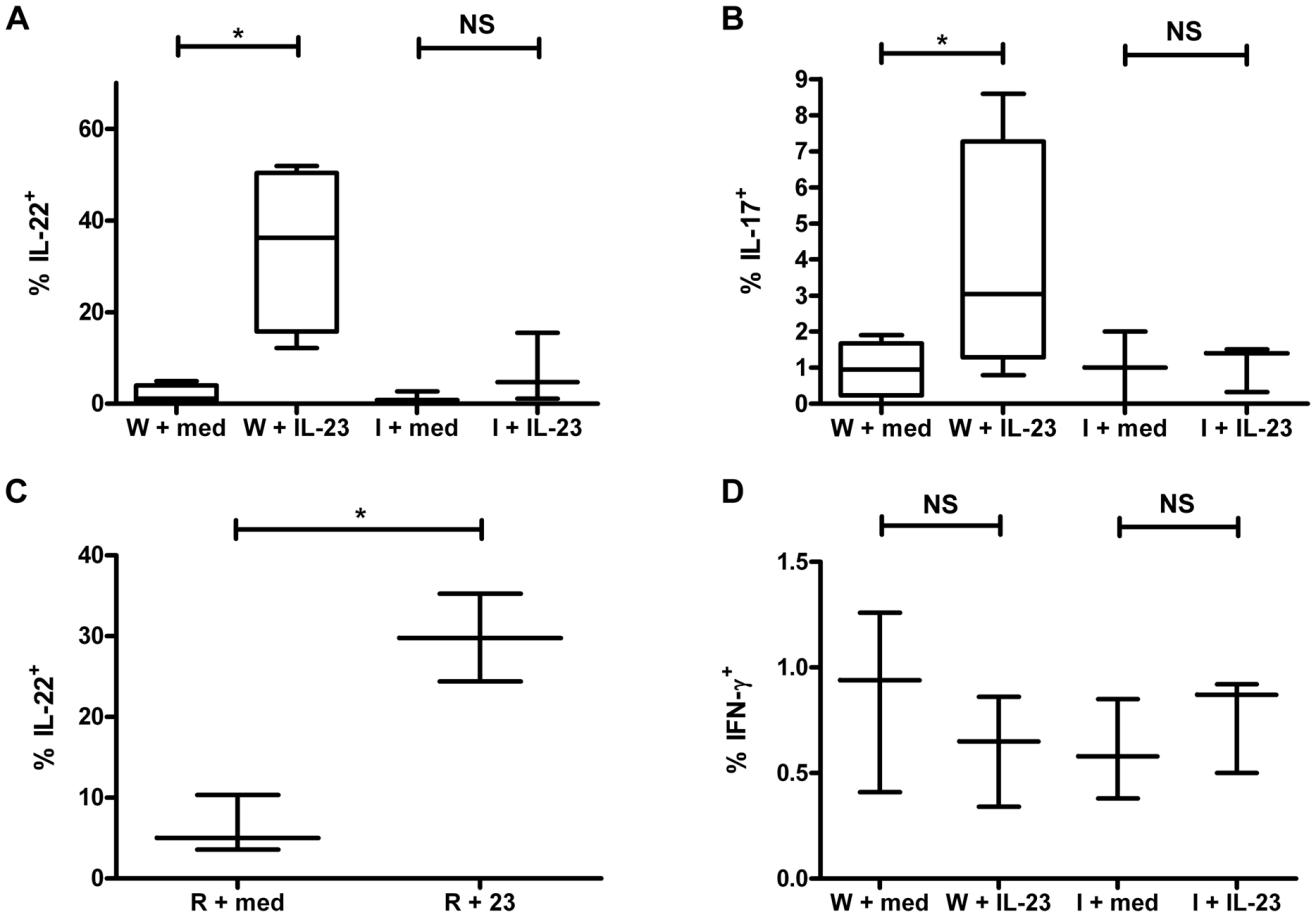
IL-1R1 is required for IL-23-stimulated IL-17 and IL-22 production by LTi-like cells ***in vitro*** **.** (A and B) Box and whiskers plot depicting percent of WT (W) or IL-1R1^−/−^ (I) colonic CD4^+^ LTi-like cells that produce IL-22 (A) or IL-17 (B). (C) Box and whiskers plot depicting percent of colonic LTi-like cells isolated from Rag1^−/−^ (R) C57BL/6J mice that produce IL-22. (D) Box and whiskers plot depicting percent of WT (W) or IL-1R1^−/−^ (I) colonic CD4^+^ LTi-like cells that produce IFN-γ. Except in (C), cells were isolated from WT (*top panels*) or IL-1R1^−/−^ C57BL/6J mice (*bottom panels*). Cells were stimulated by rIL-23 (23; *right panels*) or medium (M; *left panels*). Box and whisker plots representative of at least three independent experiments. *, *p*<0.05; NS, not significant. For box and whisker plots, line represents median, box represents 25^th^ to 75^th^ percentile range, and whiskers represent range.

Having demonstrated that cLP LTi-like cells are major innate IL-22 producers and that virtually all are IL-1R1^+^, we were interested in whether IL-1R1 signaling is important for LTi-like cell production of Th17 cytokines, as it is in other cell populations [41], [42], [43], [48]. After culturing small-intestinal and cLP cell suspensions (which include LTi-like cells, among other cell types) isolated from WT or IL-1R1^−/−^ mice with either medium alone (control) or IL-23, a known positive regulator of IL-17 [41] and IL-22 [38], [41], we measured the proportion of LTi-like cells that produced IL-22 and IL-17. As expected, IL-23-stimulated WT cLP LTi-like cells produced IL-22 in greater proportions than did unstimulated cells (Fig. 3A). However, IL-23 did not stimulate IL-1R1^−/−^ cLP LTi-like cells to produce IL-22 in greater proportions than media alone (Fig. 3A). Of note, unstimulated WT and IL-1R1^−/−^ cLP LTi-like cells produced IL-22 in comparable proportions (Fig. 3A). Qualitatively, these findings were similar with IL-17, with IL-1R1 being required for IL-23 stimulation of IL-17 as well; however, a smaller proportion of LTi-like cells produced IL-17 than IL-22 (Fig. 3B). Again, the percentages of WT and IL-1R1^−/−^ LTi-like cells producing IL-17 are similar at baseline (Fig. 3B). The pattern of small-intestinal LTi-like cell production of IL-22 and IL-17 in WT and IL-1R1^−/−^ mice was qualitatively similar to that of colonic LTi-like cells, in that IL-23 stimulation increased production of IL-22 and IL-17 in WT but not IL-1R1^−/−^ LTi-like cells (data not shown). Colonic LTi-like cells from Rag1^−/−^ mice also increased IL-22 production after IL-23 stimulation (Fig. 3C). Neither WT nor IL-1R1^−/−^ LTi-like cells produce IFN-γ either at baseline or following stimulation with IL-23 (Fig. 3D), which demonstrates that cytokine stimulation by IL-23 is not nonspecific. Taken together, these data indicate that intestinal LTi-like cells require IL-1R1 for IL-23 stimulation of IL-22 and IL-17 but that IL-1R1 is not involved in homeostatic control of either of these cytokines.

### LTi-like Cells are Important for Survival Following S. Typhimurium Infection

**Figure 4:**
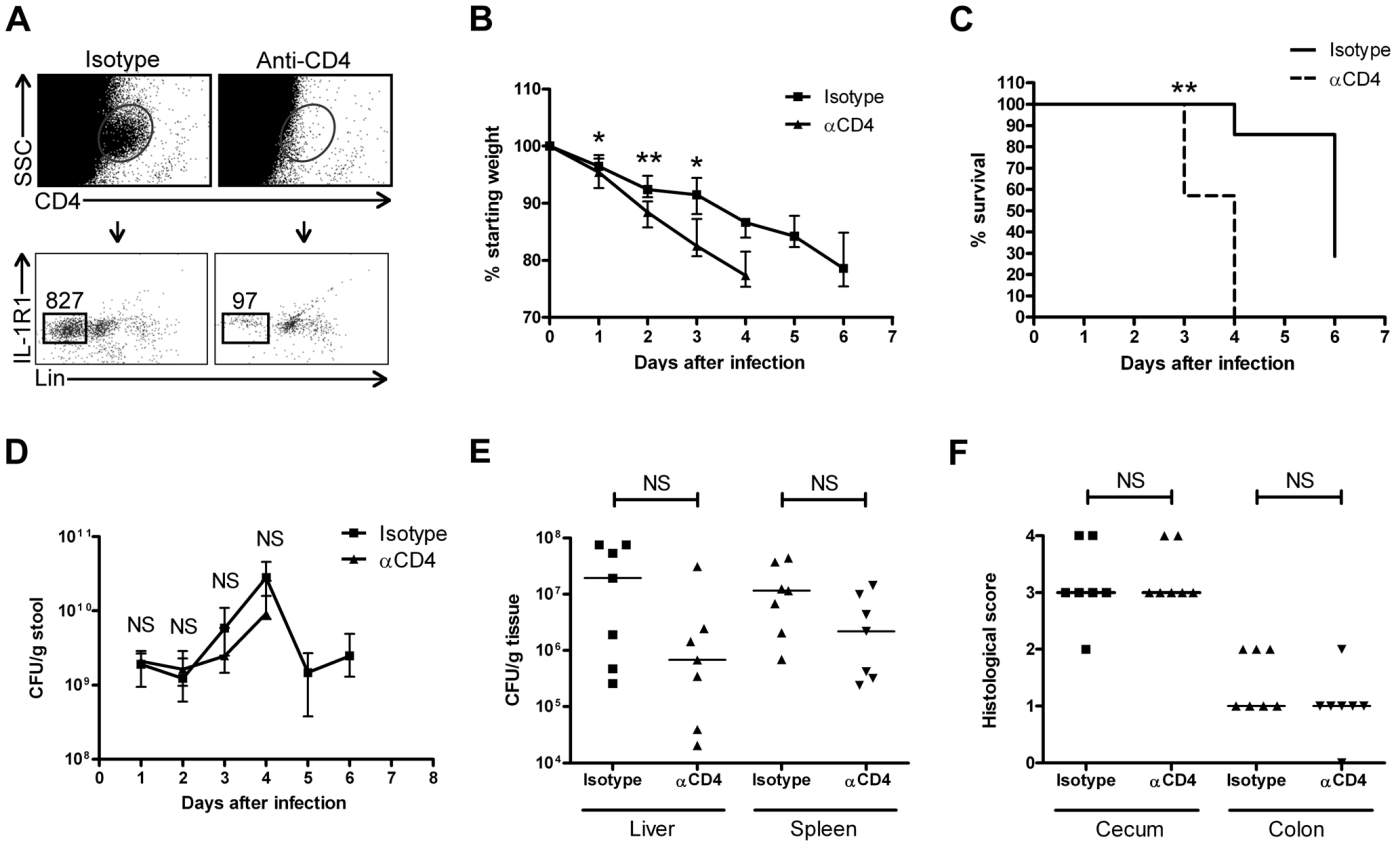
Depletion of intestinal LTi-like cells increases susceptibility to *S.* Typhimurium infection. (A) Scatter plot demonstrating depletion of colonic IL-1R1^+^ CD4^+^ Lin^−^ LTi-like cells by injection of anti-CD4 antibodies. Number of CD4^+^ cells (*top*) and LTi-like cells (*bottom*) isolated from cLP of Rag1^−/−^ C57BL/6J mice is indicated. Data shown are representative of three experiments. (B-D) Weights (normalized to starting weight) (B), survival (C), and fecal burden of *S.* Typhimurium (D) over time following *S.* Typhimurium infection in isotype control (*square*) and anti-CD4 (αCD4; *triangle*) antibody-treated mice. (E) CFUs of *S.* Typhimurium in the liver or spleen at time of death in isotype control- and αCD4-treated mice. (F) Histological scores for the cecum and proximal colon in isotype control- and αCD4-treated mice. *, *p*<0.05; **, *p*<0.01; NS, not significant.

Given that IL-22 is important in protection against *S.* Typhimurium [29], [52] and IL-1R1^+^ CD4^+^ LTi-like cells in the intestinal LP are a significant source of innate IL-22 (Fig. 2), we hypothesized that these cells play an important role in defense against *S.* Typhimurium. To test this hypothesis, we depleted CD4^+^ LTi-like cells in Rag1^−/−^ mice using a depleting anti-CD4 antibody, an approach similar to that of previous reports [25]. By day 0, injection of anti-CD4 antibodies depleted ∼90% of colonic IL-1R1^+^ CD4^+^ LTi-like cells relative to injection of an isotype control antibody (Fig. 4A). After orally infection with *S.* Typhimurium, the mice injected with an anti-CD4 antibody demonstrated accelerated weight loss (Fig. 4B) and increased mortality (Fig. 4C) compared to mice injected with an isotype control. These results clearly demonstrate that CD4^+^ LTi-like cells are critical for survival following *S.* Typhimurium infection. However, it is not clear at which step of disease pathogenesis these cells are most critical: there were no differences in stool bacterial load (Fig. 4D), bacterial dissemination to the liver or spleen (Fig. 4E), or inflammation in the cecum or proximal colon (Fig. 4F).

### Intestinal Bacteria Influence the Number of Colonic IL-1R1^+^ LTi-like Cells

**Figure 5:**
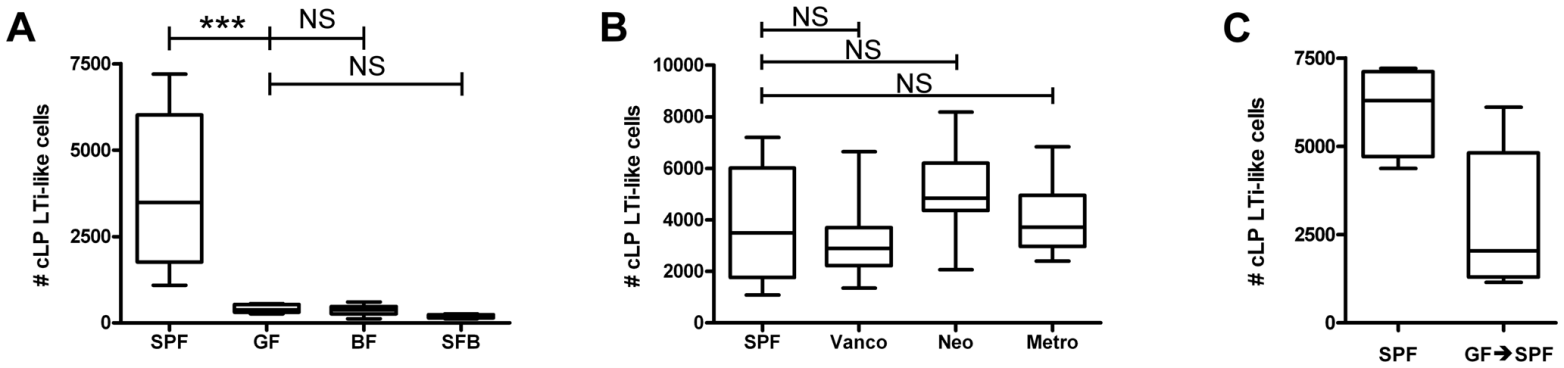
Number of colonic IL-1R1^+^ CD4^+^ LTi-like cells depends on the gut flora. (A) Number of IL-1R1^+^ CD4^+^ LTi-like cells in the cLP of Swiss-Webster mice that were either SPF, GF, monocolonized with *B. fragilis* (BF), or monocolonized with segmented filamentous bacteria (SFB). *n* = 8−10. (B) Number of IL-1R1^+^ CD4^+^ LTi-like cells in the cLP of SPF Swiss-Webster mice treated for five weeks with either vancomycin (vanco), neomycin (neo), or metronidazole (metro). N = 8−10. (C) Number of IL-1R1^+^ CD4^+^ LTi-like cells in the cLP of Swiss-Webster mice that were either SPF or born GF and co-housed at weaning age with SPF mice (GF→SPF). *n* = 4−5. *, *p*<0.05; ***, *p*<0.001; NS, not significant. For box and whisker plots, line represents median, box represents 25^th^ to 75^th^ percentile range, and whiskers represent range.

The effect of the intestinal microbiome on the development and function of the intestinal mucosal immune system is becoming increasingly recognized [49]. Although small-intestinal LTi-like cell numbers are not dependent on the microbiome [26], we sought to examine whether the microbiome affects cLP LTi-like cells. We found that GF mice have a lower absolute number of cLP IL-1R1^+^ CD4^+^ LTi-like cells than SPF mice (Fig. 5A), demonstrating that cLP LTi-like cell numbers depend on host-bacterial interactions. To investigate whether these interactions require specific bacteria or merely the presence of any bacteria (perhaps through general microbe-associated molecular patterns such as Toll-like receptors), we analyzed mice monocolonized with *B. fragilis* or SFB, prototypical Gram-negative and Gram-positive organisms, respectively, that are known to have significant immunomodulatory effects on the intestinal immune system [6], [8], [9]. Neither organism alone increased the number of cLP LTi-like cells above that of GF mice (Fig. 5A), revealing that cLP LTi-like cells depend on specific bacteria other than the two organisms tested.

To attempt to understand which microbe(s) is required for generation of cLP LTi-like cells, we treated three groups of SPF mice with a single antibiotic for ∼5 weeks beginning at the time of weaning. Each antibiotic used targets a different class of bacteria: vancomycin, neomycin, and metronidazole affect Gram-positive, Gram-negative, and anaerobic bacteria, respectively. Gram staining of fecal samples verified depletion of the relevant bacterial populations for the vancomycin- and neomycin-treated mice (data not shown). Surprisingly, none of the antibiotic treatments had an effect on the absolute number of cLP IL-1R1^+^ CD4^+^ Lin^−^ LTi-like cells (Fig. 5B), suggesting that there is either redundancy between bacterial classes or that relevant host-bacterial interactions occur prior to weaning. To begin to investigate the latter possibility, we “conventionalized” germ-free mice at weaning (∼3 weeks old) by co-housing them with SPF mice, analyzing the cLP LTi-like cell population at 6–8 weeks of age. Compared to SPF mice, conventionalized mice had fewer colonic LTi-like cells (Fig. 5C). Taken together, these results indicate that the intestinal microbiota plays an important role in the generation of IL-1R1^+^ CD4^+^ LTi-like cells and that early bacterial exposure may be necessary to induce normal numbers of cLP LTi-like cells.

## Discussion

We focus in this paper on a subset of cells within the larger category of group 3 ILCs, namely the CD4^+^ LTi-like cells. We have demonstrated that CD4^+^ LTi-like cells nearly universally express IL-1R1. These cells are important producers of IL-22 in both healthy and inflammatory conditions, and they require IL-1R1 for IL-23 stimulation of IL-22 and IL-17, highlighting a signaling pathway important for the activation of these LTi-like cells. Likely as a result of this IL-22 production, LTi-like cells enhance survival following *S.* Typhimurium infection. Finally, we determined that the microbiota is required for the accumulation of cLP IL-1R1^+^ CD4^+^ LTi-like cells.

Group 3 ILCs are a major source of innate IL-22 [26], [30], [53]. There is not entirely clear whether ILC-produced IL-22 is constitutive [44] or requires induction [17], [33], [51]. Previous reports have demonstrated that IL-1R1 signaling is required for steady-state IL-22 expression in small-intestinal ILCs and that NKp46^−^ RORγt^+^ ILCs are stimulated by IL-23 to produce IL-22 [44]. We have extended this work by demonstrating that IL-1R1 is essential for IL-23-induced IL-22 production by LTi-like cells in the cLP and found that there is very little steady-state production of IL-22 by cLP LTi-like cells. The mechanism by which IL-1 and IL-23 signaling synergize in ILCs is unclear. In CD4^+^ T cells, IL-1R1 signaling enhances RORγt expression [44] and thus promotes differentiation [41] and maintenance [34] of Th17 cells. In ILCs, though, IL-1 does not appear to promote RORγt expression [44]. Research on IL-1 and IL-23 signaling in T cells has identified several molecules other than RORγt involved in their synergy, including aryl hydrocarbon receptor, nuclear factor-κB, phosphatidylinositol 3-kinase, novel protein kinase C, and signal transducer and activator of transcription 3 [54], [55], [56]. Additional work is needed to determine whether any of these pathways are involved in the IL-1/IL-23 synergy seen with LTi-like cells.

The importance of IL-1 in driving LTi-like cell production of IL-22 has physiologic significance. IL-1 is an acute phase reactant that can be produced by innate immune, epithelial, and dying cells, and as its production does not require antigen presentation it can be produced rapidly [39]. Interestingly, IL-1 signaling does not appear to play a major role in inducing IL-22 production under homeostatic conditions (see Fig. 3A and 3B). Like IL-1, IL-22 is most important in the early phase of infections [25], [57] given that its primary mechanism of protection is believed to involve promotion of tissue healing [58] that is important for local containment of disease as opposed to controlling systemic disease. Our findings that LTi-like cell-depleted mice died earlier but had a similar extent of disseminated disease at time of death and that fecal burden of *Salmonella* was identical in the presence and absence of LTi-like cells are consistent with this hypothesis. The fact that we observed no difference in histological scores of the cecum and colon may be due, in part, to the time point selected; we were somewhat constrained in when we could assess histology given that mice injected with anti-CD4 antibodies died rapidly from the infection. Presumably, the loss of LTi-like cell-produced IL-22 led to poor tissue healing and earlier bacterial dissemination that led to accelerated weight loss and earlier death.

There are some limitations with the *Salmonella* infection and LTi-like cell depletion model. Although *Salmonella* affects the entire intestine–not just the colon [59]–we focused our investigations on the colon. Given that small-intestinal and colonic LTi-like cells had qualitatively similar cytokine production patterns (Fig. 3), small-intestinal LTi-like cells probably have a similar function in *Salmonella* infection as cLP LTi-like cells. While we demonstrated that colonic LTi-like cells were nearly completely depleted with anti-CD4 antibodies, we did not specifically assess small-intestinal LTi-like cells, though we presume that these were similarly depleted. An additional limitation of this model is that injection of Rag1^−/−^ mice with anti-CD4 antibodies is not specific for depletion of LTi-like cells and may affect other innate cell populations, such as DCs. Although phagocytosis of bacteria by DCs drives *Salmonella* dissemination [59], CD4^+^ DCs constitute a very minor portion of the entire DC population [60]. Moreover, this anti-CD4 depletion approach is an accepted method to investigate the role of LTi-like cells in enteric infections [25]. However, we cannot exclude the possibility that another CD4^+^ cell population was also important for survival following *S.* Typhimurium infection.

There is controversy about the role of the microbiome in determining ILC numbers [51]. Eberl and colleagues previously reported that the number of small-intestinal RORγt^+^ NKp46^−^ LTi cells is not significantly different in GF mice compared to SPF mice, demonstrating that the microbiota does not impact the ontogeny of these cells [26]. Cell numbers of a different class of ILCs, small-intestinal NKp46^+^ RORγt^+^ cells, have been varyingly reported to be normal [44] or decreased [17], [33] in GF mice compared to SPF mice. At first glance, these findings may appear to conflict with our conclusion that the microbiota impacts colonic CD4^+^ LTi-like cell numbers (see Fig 5A). However, to our knowledge, this is the first report comparing colonic LTi-like cell numbers in GF and SPF mice. It is well known that the microbiota can have immunomodulatory effects restricted to a particular anatomical compartment. For example, *Clostridium* species induce regulatory T cells in the colon but not in the small intestine [5], yet-unspecified bacteria downregulate colonic but not ileal natural killer T cells [10], and intestinal colonization with *B. fragilis* increases Th1 cell numbers in the spleen but not in the mesenteric lymph nodes [61]. The mechanism underlying these compartment-related differences in cell numbers is unclear but may be related to differences in the specific bacterial microenvironment [62]. In addition to compartment-related differences, it is important to emphasize that the population of cells studied in this paper is a subset of the larger class of RORγt^+^ NKp46^−^ ILCs [19], and conclusions about the larger group may not apply to this smaller subset.

Similarly, the exact role of the microbiome in influencing colonic lymphoid tissue development is not entirely clear. Mebius and colleagues showed that GF mice have normal numbers of colonic solitary intestinal lymphoid tissues (SILTs) [37], which argues against a role of the microbiome in driving clustering of LTi cells in the colon. However, they found that a greater proportion of the SILTs in GF mice were immature [37]. In contrast, Eberl and colleagues reported greatly decreased number and maturation of colonic SILTs in GF mice [22]. The reasons for this discrepancy are unclear. Of note, neither paper reported LTi cell number. Nonetheless, taken together with these earlier publications, our findings suggest that the diminished number of the IL-1R1^+^ CD4^+^ LTi cells present in GF mice may be sufficient to induce lymphogenesis under particular circumstances, but that full maturation of SILTs may either require a greater number of these cells or additional interactions with the microbiota.

Our attempts to identify a specific class of organism responsible for colonic LTi-like cell numbers were unrevealing in that antibiotic treatment of adult mice with three antibiotics chosen for their specific spectra of activity does not affect LTi-like cell counts. In addition, bacterial colonization of adult GF mice does not lead to full restoration of LTi-like cell numbers. In contrast, Th17 cell numbers can be rapidly and completely restored in conventionalized adult GF mice [7]. The discrepancy in these observations may be due to increased redundancy in the populations of bacteria involved in induction of LTi-like cells than in that of T cells. Alternatively, an intriguing possibility is that–similar to natural killer T cells [10]–there is a critical window period after birth in which LTi-like cell development is bacteria-dependent, after which subsequent perturbations in the microbiota have little effect on cell numbers. In this study, the antibiotic-treated mice were raised under SPF conditions until weaning; moreover, we “conventionalized” GF mice at three weeks of age, which may be beyond the critical window period. Although our data only suggest and do not definitively demonstrate that there is such a window period, one can envision a teleological imperative for innate immune cells–which constitute the front line of defense against infection [25]–to be programmed early in life and not be subject to fluctuations in the microbiota [10]. On the other hand, cells of the adaptive immune system, which specifically recognize a greater diversity of antigens, need to be able to respond to a changing environment; accordingly, these cell numbers vary dynamically with the microbiota [2], [6], [7]. Future studies will further delineate the effect of the timing of microbial exposure on LTi-like cells and other ILCs, with the aim of identifying specific bacteria that influence these cell types.

## Materials and Methods

### Mice

WT, IL-1R1^−/−^, and Rag1^−/−^ C57BL/6J mice were purchased from Jackson Laboratories. SPF Swiss Webster mice were initially purchased from Taconic Farms and subsequently bred and maintained and conventional housing. GF Swiss-Webster mice were initially purchased from Taconic Farms and subsequently bred and maintained in sterile isolators at Harvard Medical School using sterile food, water, and bedding. Swiss-Webster were used only for experiments involving comparison of intestinal flora (i.e. Fig. 5). C57BL/6J mice were used for all other experiments. GF mice were confirmed to be germ-free by weekly cultures of fecal samples in aerobic and anaerobic environments. *B. fragilis*- and SFB-monocolonized Swiss-Webster mice were generated as previously described [8], [49]. All commercially obtained mice were maintained in our animal facility for at least one week after arrival before being used for experimentation. All procedures with animals were performed according to the Harvard Medical School Office for Research Subject Protection guidelines and were approved by the Harvard Medical Area Standing Committee on Animals.

### Cell Isolation

Intestinal intraepithelial lymphocytes and lamina propria lymphocytes were isolated as previously described [50]. Briefly, mouse intestines were flushed clean of feces, inverted, and stirred in 3% FBS, 1 mM EDTA, and 0.015% DTT in PBS at 37°C for 30 minutes to extract intraepithelial lymphocytes and epithelial cells into the solution. The remaining tissue was digested with 20 mL RPMI 1640 containing 5% FBS, 30 mg collagenase (Gibco), and 10 mg dispase (Gibco). The resulting suspension was passed through a strainer (100 µm, Fisher Scientific) and lamina propria lymphocytes were isolated from this suspension using centrifugation at 300g for 10 minutes.

### Flow Cytometry

Cells were resuspended in FACS buffer (PBS containing 10 mM EDTA, 15 mM sodium azide, and 1% bovine serum albumin). All antibodies were purchased from Biolegend, except for antibodies against mouse IL-22, CD3, and CD11c, which were purchased from BD Biosciences. Cells were labeled with the appropriate antibodies following manufacturer directions in the presence of 1 µg/mL anti-mouse CD16/CD32 (Biolegend) for 30 minutes at 4°C. Cells were then washed twice, resuspended in FACS buffer, and analyzed with a FACSCalibur system (BD Biosciences).

### Intracellular Staining

Cells were first stained with anti-mouse CD16/CD32 and fluorophore-conjugated antibodies against surface antigens for 30 minutes at 4°C. The cells were permeabilized and fixed with Cytofix/Cytoperm solution (100 µL; BD Biosciences) at 4°C overnight. After two washes with Perm/Wash buffer (BD Biosciences), cells were labeled with fluorescent-conjugated antibodies against intracellular antigens for 30 minutes at 4°C, washed twice with a Perm/Wash buffer (BD Biosciences), resuspended in FACS buffer, and analyzed on a FACSCalibur system (BD Biosciences).

### DSS Colitis

Mice were fed 3% DSS (molecular weight 36,000–50,000; MP Biomedicals) *ab libitum* in their drinking water. Weight was measured daily and mice were euthanized when they lost 20% of their starting weight or appeared moribund.

### In vitro Cytokine Stimulation

Isolated lamina propria lymphocytes were resuspended in PBS and passed through a column containing approximately 20 mg of glass wool per colon. These cells were then placed in RPMI 1640 (Invitrogen) containing 10% FBS (Gibco). rIL-23 (Biolegend) or medium was added as indicated, and the cells were cultured at 37°C with 5% CO_2_. After 12–16 hours, brefeldin A (Sigma-Aldrich) was added to a final concentration of 10 ng/mL, and cells were analyzed by flow cytometry 5 hours later.

### *Salmonella* Studies

Three days and one day prior to infection with *S.* Typhimurium (strain SL1433; a kind gift from David Relman), 6–8 week old mice were injected intraperitoneally with 200 µg anti-CD4 antibody (GK1.5 clone) or isotype control. One day prior to infection, the mice also received 2 mg streptomycin (Sigma) by oral gavage. The following day, mice were orally gavaged with ∼5×10^3^ CFU *S.* Typhimurium. Fecal pellets were collected and body mass measured daily. Fecal samples were homogenized in LB broth (Fisher) and plated on LB agar (Fisher); LB broth and agar contained 200 µg/mL streptomycin (Sigma). Colonies were counted 16–24 hours later. Cultures of the stool and organs from uninfected mice did not grow colonies. Mice were euthanized after losing 20% of their starting weight or when they appeared moribund. The colon and cecum were harvested for histology at time of euthanasia. The liver and spleen were also harvested at this time and homogenized and plated as described above for fecal samples.

### Histopathology

Cecal and proximal colon samples were fixed in Bouin’s solution (Ricca Chemical Company), and thin sections stained with hematoxylin and eosin were prepared by the Rodent Histopathology Core of the Dana Farber/Harvard Cancer Center. Extent of inflammation was assessed by a pathologist who had no knowledge of the experimental design. Slides were scored for inflammation, ulceration, and edema as follows: 0, no disease; 1, mild disease; 2, moderate disease; 3, severe disease; 4, very severe disease.

### Antibiotic Administration

Vancomycin (0.5 g/L; Sigma-Aldrich), neomycin (1 g/L; MP Biomedicals), or metronidazole (1 g/L; MP Biomedicals) was added to the drinking water of mice starting following weaning (around 3 weeks old). Mice were maintained on the antibiotic until sacrifice at around 8 weeks of age.

### Statistics

In the survival curve in Fig. 4C, statistical significance was determined using the log-rank test. In Fig. 3, paired *t* tests were used. For all other comparisons, statistical significance was determined using the Mann-Whitney test. For all comparisons, *p* values <0.05 were considered significant.
